# The 36-Item Yoni Task: Normative Data for the Clinical Assessment of Theory of Mind

**DOI:** 10.3390/jcm13216334

**Published:** 2024-10-23

**Authors:** Sara Isernia, Federica Rossetto, Antonella Marchetti, Francesca Baglio

**Affiliations:** 1 IRCCS Fondazione Don Carlo Gnocchi ONLUS, 20148 Milan, Italy; sisernia@dongnocchi.it (S.I.); fbaglio@dongnocchi.it (F.B.); 2 Research Unit on Theory of Mind, Department of Psychology, Università Cattolica del Sacro Cuore, 20123 Milan, Italy; antonella.marchetti@unicatt.it

**Keywords:** social cognition, theory of mind, neurocognitive assessment, validation, rehabilitation, normative data

## Abstract

**Background:** The evaluation of Theory of Mind (ToM) in the clinical setting remains limited due to the lack of valid instruments for a quick and reliable assessment. In this study, the psychometric properties of the Yoni-36 task were tested, and correction grids, equivalence scores, and normative data were computed. **Methods**: In total, 318 healthy subjects (169 females; mean age = 42.22 ± 18.11 years; mean education = 15.01 ± 3.43 years) were enrolled and administered the Yoni-36 task. **Results**: Statistical analysis showed good-to-high internal consistency, split-half reliability, and discrimination ability (especially for the second-order items) of the Yoni-36 task. Regression models highlighted the predictive role of age and education on second-order, affective, cognitive accuracy, and response time scores. Education influenced the first-order ToM response time score as well. Based on β values of the regressions, raw scores of the Yoni task were adjusted for sex, age, and education, and correction grids were computed. The equivalent scores indicated an accuracy index score < 0.69 and a response time index score < 0.83 as cut-off points for ToM competence. Normative data reported a mean accuracy and response time index score of 0.90 ± 0.11 and 0.91 ± 0.05 in the Italian population, respectively. **Conclusions**: The Yoni-36 proved to be a valid instrument for a quick and reliable ToM assessment, suitable to be included in neuropsychological batteries and to be used in routine clinical practice.

## 1. Introduction

Social cognition (SC) refers to neurocognitive processes involved in the capacity “*to learn about the world from others, to learn about other people, and to create a shared social world*” by processing and interpreting social signals [1]. Recently, awareness of the role of social skills in mental health and well-being has grown exponentially, with a flourishing research interest in social cognitive functions [2]. From a clinical perspective, the latest edition of the American Psychiatric Association’s Diagnostic and Statistical Manual for Mental Disorders, Fifth Edition, Text Revision (DSM-5-TR) describes SC as a core component of neurocognitive functioning along with attention, executive functions, memory, language, and visuospatial abilities. Even more relevant, SC disturbances contribute to the diagnostic criteria for numerous pathological conditions. Accordingly, recent evidence highlighted social cognitive disturbances as a signature of the cognitive phenotype in neurological, neurodegenerative, and psychiatric conditions [3,4,5,6,7]. For example, SC impairment can be a disabling symptom following acute brain injuries, including traumatic brain injury or stroke [6]. Moreover, altered SC competency has been described in the early stages of several chronic neurological disorders, such as the behavioral variant of frontotemporal dementia, Alzheimer’s disease, Parkinson’s disease, and multiple sclerosis [4,5]. Based on the role of SC in the diagnostic process of the aforementioned pathological conditions, a systematic evaluation of social abilities should be incorporated into the preliminary standard neurocognitive evaluation and in the subsequent routine clinical follow-up.

A core SC competency is Theory of Mind (ToM), which is the ability to understand mental states, such as intentions and feelings, of oneself and others [2]. As a multidimensional psychological construct, ToM allows to make inferences both on cognitive mental states, such as beliefs and thoughts (cognitive ToM component), and affective mental states, such as desires and emotions (affective ToM component) [2,8,9,10]. Evidence of these distinct dimensions arose from neuroimaging studies, which reported the role of different brain regions mediating cognitive and affective ToM, such as the dorsomedial, dorsolateral, and ventromedial, orbitofrontal portions of the prefrontal cortex, respectively [11]. Moreover, understanding mental states relies on a hierarchical level of meta-representational attribution, which includes first- and second-order ToM. From a developmental perspective, ToM capacities appear early in infancy, around eighteen months, with the development of shared attention and declarative pointing [12]. By the age of two, a solid understanding of others’s desires and mental states begins to emerge [13]. Then, by ages three to four, children recognize that people have different beliefs from their own [14]. First-order ToM represents the first developmental milestone in the understanding of another’s mental state. This competence allows people to represent themselves and understand mental states of others (e.g., “*he/she thinks that…*”) and develops around four years of age [9,14,15,16,17]. The second level of recursive thinking (second-order ToM) begins around eight years of age, especially in the context of formal schooling, and requires individuals to represent the mental states of others with respect to different individuals (e.g., “*he/she thinks that he/she thinks that…*”) [9,14,15,16,17], with a substantial impact on intra- and inter-personal competence [18].

Importantly, a partial dissociation between first- and second-order affective and cognitive ToM has been observed in healthy aging [17,19] and neurological and neuropsychiatric conditions including localized brain lesions [20], schizophrenia [21], multiple sclerosis [22,23,24], Parkinson’s disease [25,26,27], Alzheimer’s disease [28], and Mild Cognitive Impairment [27,29]. Especially, greater impairment in affective compared to cognitive ToM has been reported in multiple sclerosis [30], while second-order ToM tasks seem to be first affected in age-related pathological processes [31,32,33,34].

Given the relevance of a multidimensional assessment of ToM in the clinical setting and in line with its multicomponent nature, ToM needs a multifaceted assessment. However, ToM measures are traditionally conceived to evaluate components or levels of ToM and are extremely varied in their assessment, including static vs. dynamic and the use of visual vs. non-visual stimuli. For example, the False Belief task is based on written stories to assess cognitive first-order [14,35] and second-order ToM [15]. Similarly, the Faux Pas Recognition test [24,36] and the Strange Stories test [37] present complex real-life social situations (e.g., white lie, double bluff, irony, misunderstanding, metaphor) in written stories to evaluate ToM without considering the level of recursive thinking. Differently, the Story-based Empathy task [38] and the Reading the Mind in the Eyes test (RMET [39]) involve visual static stimuli for assessing affective and cognitive components of ToM. Finally, other tests show ecological dynamic stimuli (multimedia content) resembling complex real-life social interactions to assess both affective and cognition ToM and other social cognition abilities (e.g., emotion recognition, moral cognition), or social cognition in a broader sense, such as the The Awareness of Social Interest Test (TASIT [40]), the Edinburgh Social Cognition Test (ESCoT [41,42,43]), and the Movie for the Assessment of Social Cognition (MASC [44]). However, their use is hardly suitable in different cultures due to the presence of verbal dialogs, gestures, prosody, and/or culture-dependent social norms. Three relevant issues regarding ToM assessment have emerged: first, most of the adopted measures evaluate only a subset of ToM domains (affective and/or cognitive ToM components and/or first and/or second ToM levels), making it difficult to obtain a global ToM assessment. Second, normative data are available for only a limited number of these measurements (see, for example, [38,41,45,46]). Finally, ToM tools, as well as most SC measures, are influenced by and dependent on cultural background and ethnicity, making it crucial to conduct population-based validation studies [47].

The Yoni task [8,27,48] is a digital test allowing to evaluate comprehension of mental states (affective mental states, i.e., emotions, and cognitive mental states, i.e., thoughts). The stimuli are static visual-spatial-colored cartoon-like pictures that are used to assess mental state understanding based on facial expression and direction of gaze. Our recent works [48,49] provided two short versions of the task (the 48-item version, the Yoni-48 task, and the 36-item version, the Yoni-36 task), developed from the 98-item version, suitable to be adopted for first-level neuropsychological assessment. Results from the validation study of the 48-item version of the Yoni task [49] supported its validity and reliability, in line with the earlier study on the 98-item version. Moreover, correction grids and normative data have been provided for the Italian population to enhance the application of the tool in the clinical context. However, limited time for most clinical visits has motivated the development of a ToM assessment that can be included as part of a comprehensive neuropsychological assessment battery. As such, the aim of the present study consists of providing correction grids and normative data for an even shorter version of the test, the Yoni-36 task [48,49]. This latter version of the test maintains the multidimensional evaluation of ToM in a shorter administration time than the Yoni-48 task, with the further advantage of being well-balanced in terms of affective, cognitive, and first- and second-order ToM items (ToM components and levels). In fact, the Yoni-48 task score is based on a higher number of ToM second-order items than first-order ones, which allows the test to be more challenging but, simultaneously, leads to lengthy administration time. Therefore, the adoption of the more concise version could be an option due to time constraints or for ToM screening purposes.

This study aims to substantially contribute to the field of neurocognitive assessment. Specifically, it evaluates the psychometric properties of the Yoni-36 task, intending to offer a validated tool for the multidimensional evaluation of ToM, which is now regarded as one of the fundamental pillars of neurocognitive functioning, even in clinical settings. Specifically, these research goals were: (1) to test the validity of the Yoni-36 task (item discrimination ability) and its inter-item reliability (internal consistency and split-half reliability), and (2) to provide normative data and equivalent scores for the Italian population. Validation of the Yoni-36 task will offer a quick and reliable tool for assessing ToM in the diagnostic process within routine clinical practice, ensuring a comprehensive evaluation of ToM components and levels in shorter administration times, as necessitated by time constraints of the clinical setting.

## 2. Materials and Methods

### 2.1. Design and Participants

This is a prospective cross-sectional study conducted at the IRCCS Don Gnocchi Foundation of Milan, in line with the Declaration of Helsinki, after approval of the Don Gnocchi Ethics Committee. All the participants read and signed the written informed consent sheet before taking part in this research.

Subjects were enrolled in this study at the IRCCS Don Gnocchi Foundation of Milan. They were students, staff of the clinic, volunteers, and patients’ caregivers. Participation in this study was voluntary, and subjects did not receive compensation for taking part in this research. They were enrolled in this study by a researcher (a psychologist). Recruitment was carried out by information flyers at the clinic or by word of mouth. The following inclusion/exclusion criteria were considered: age ≥ 18; years of education ≥ 5; absence of neurological, major psychiatric, organic conditions, visual or auditory disability, or pharmacological treatment known to affect test performance, as assessed by an ad-hoc questionnaire.

### 2.2. Materials and Procedure

All recruited subjects were invited to take part in an individual session lasting about 10 min. During the session, a psychologist collected participants’ demographics and administered the computerized version of the Italian 36-item Yoni task (Yoni-36 task). 

**Figure 1:**
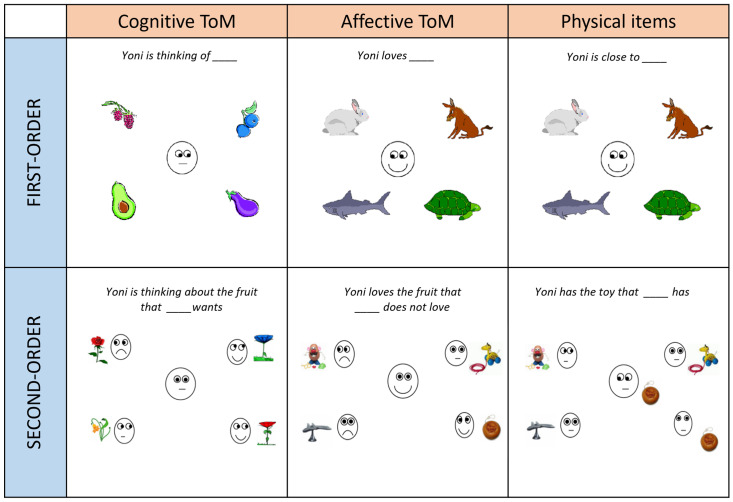
Sample of items from the Yoni task: first- and second-order cognitive and affective mental inference and physical (control) items. Adapted from [27].

The Yoni-36 task is the short version of the Italian Yoni task [48], a multidimensional measure of the ToM ability. It is composed of 36 items in total: 32 ToM and 4 control (physical) stimuli. The 32 ToM stimuli include 16 first-order (8 affective and 8 cognitive) and 16 second-order (8 affective and 8 cognitive) items. The stimuli are static visual-spatial colored cartoon-like pictures in which a face (Yoni) is placed at the center of the screen and is surrounded by four elements (fruits, animals, means of transport, or faces). Yoni’s facial expressions and gaze suggest what Yoni loves or thinks. To complete the task, subjects are instructed to click as fast as possible on the element to which Yoni refers, based on the instruction reported at the top of the screen (*“Yoni loves…”, “Yoni is thinking of…”, “Yoni is thinking about the fruit that…wants”*) (see Figure 1). The maximum time available to answer each item is 60 s. Two scores per item are recorded: accuracy and response time. To compute accuracy, there is only one correct response per item, scored 0 or 1, while each item’s response time ranges from 0 to 60 (seconds). Raw total accuracy scores are computed by summing up first-order, second-order, cognitive, and affective items separately (range 0–16). Raw response time scores are computed by averaging first-order, second-order, cognitive, and affective items separately (range 0–60). Then, after adjusting total scores (see Results), composite scores for accuracy (ACC) and response time (RT), both ranging from 0 to 1 (a higher score indicates greater performance), are computed (see the “Adjustment of raw scores of the Yoni-36 task” paragraph). Moreover, a score indicating the balance between the cognitive and affective ToM performance is also calculated (CA, see the “Adjustment of raw scores of the Yoni-36 task” paragraph).

### 2.3. Statistical Analysis

For statistical analysis, JASP (JASP Team 2020; version 0.16.1) and IBM SPSS Statistics (version 28.0.1.1) were used.

*Participants’ demographics:* Frequencies, means, and standard deviations were reported to describe demographic characteristics.
*Yoni-36 task validity*: To assess discrimination ability for each item, participants were divided into two sub-groups based on the median of the Yoni-36 total raw score (high performance-group: score ≥ median; low-performance group: score < median), and the mean discrimination ability (Ebel’s D) effect size (h) was computed. Discrimination ability was also evaluated with the dichotomous Rash model (Item Response Theory): information-weighted mean square statistic and outlier-sensitive mean square statistic.
*Yoni-36 task reliability*: McDonald’s ω, Cronbach’s α, Guttman’s λ^2^, and Guttman’s λ^6^, and Spearman-Brown ϱ_SP_ split-half reliability were reported to test inter-item reliability.
*Yoni-36 task correction grids*: The effect of sex, age, and education on Yoni-36 raw total scores was tested by running simultaneous regression models. Then, regression coefficients and the means of the demographic variables were used to algebraically determine the adjustment of raw scores (see Results for details).
*Yoni-36 task normative data and equivalent scores extraction*: Mean, median, standard deviation, minimum and maximum value, 25th–75th percentiles were reported for each Yoni-36 adjusted score and total score (first-order, second-order, affective, cognitive adjusted score, ACC, RT, CA_A_, CA_RT_). Then, Capitani and Laiacona’s [50,51] indications were followed to extract equivalent scores for ACC and RT indexes.

## 3. Results

### 3.1. Participants

318 subjects participated in this research. Table 1 reports the number of subjects included in this study stratifying per sex, age, and years of education groups. 

### 3.2. Internal Consistency and Split-Half Reliability

The Yoni-36 task showed good-to-high internal consistency and split-half reliability. Table 2 reports inter-items and split-half reliability indexes for the Yoni-36 total, second-order, first-order, affective, and cognitive scores.

### 3.3. Item Discrimination Ability

Based on the median of the Yoni-36 total raw score (median = 30), two groups were defined (high-performance group, score ≥ 30, n = 186; low-performance group, score < 30, n = 132), and the mean item discrimination ability (Ebel’s D) effect size (h) was computed for all mental items, first-order mental items, and second-order mental items, which respectively showed a moderate (h = 0.65 ± 0.32), low (h = 0.37 ± 0.12), and high (h = 0.93 ± 0.16) effect size (see Appendix A, Table A1 for details).

The Dichotomous Rash model suggested a consistent measurement of the unidimensional construct of the task (mean square infit = 0.99 ± 0.08, mean square outfit = 1.03 ± 0.50). Specifically, the first-order items showed a mean square infit = 0.98 ± 0.13 and a mean square outfit = 0.94 ± 0.43, while the second-order items showed a mean square infit = 1.01 ± 0.13 and a mean square outfit = 0.95 ± 0.25.

### 3.4. Demographics Influence on the Yoni-36 Task

The simultaneous multiple regression model reported a predictive effect of age on second-order, affective, and cognitive accuracy and RT scores. Similarly, the level of education showed an effect on RT second-order, affective, and cognitive RT scores and on second-order and cognitive accuracy scores. No effect of demographics on first-order accuracy, while a predictive effect of education was observed for the RT score (Table 3).

### 3.5. Adjustment of Raw Scores of the Yoni-36 Task

Based on the regression coefficient and the mean scores of the sample, the following formulas were computed to adjust the Yoni-36 task raw scores:

$$first\;order\;accuracy\;adjusted\;score=raw\;score+0.01\times \left(age-42.22\right)-0.02\times \left(education-15.01\right)+0.04\times \left(sex-0.53\right)$$

$$second\;order\;accuracy\;adjusted\;score=raw\;score+0.03\times \left(age-42.22\right)-0.20\times \left(education-15.01\right)+0.01\times \left(sex-0.53\right)$$

$$affective\;accuracy\;adjusted\;score=raw\;score+0.01\times \left(age-42.22\right)-0.07\times \left(education-15.01\right)+0.02\times \left(sex-0.53\right)$$

$$cognitive\;accuracy\;adjusted\;score=raw\;score+0.02\times \left(age-42.22\right)-0.16\times \left(education-15.01\right)+0.04\times \left(sex-0.53\right)$$

$$first\;order\;RT\;adjusted\;score=raw\;score-0.01\times \left(age-42.22\right)+0.19\times \left(education-15.01\right)-0.25\times \left(sex-0.53\right)$$

$$second\;order\;RT\;adjusted\;score=raw\;score-0.08\times \left(age-42.22\right)+0.28\times \left(education-15.01\right)-0.02\times \left(sex-0.53\right)$$

$$affective\;RT\;adjusted\;score=raw\;score-0.04\times \left(age-42.22\right)+0.29\times \left(education-15.01\right)-0.21\times \left(sex-0.53\right)$$

$$cognitive\;RT\;adjusted\;score=raw\;score-0.06\times \left(age-42.22\right)+0.19\times \left(education-15.01\right)-0.02\times \left(sex-0.53\right)$$

Males were coded as 1 and females as 0 to compute the adjusted scores.

Total scores of the Yoni-36 task were obtained through the following formula:

$$ACC=\frac{first\;order\;adjusted\;ToM\;accuracy\;score+second\;order\;adjusted\;ToM\;accuracy\;score}{N_{i}}\;$$

with Ni referring to the total number of items (N_i_ = 32).

$$RT=1-\frac{\frac{first\;order\;adjusted\;ToM\;response\;time\;score-{RT}_{min}}{{RT}_{i}}+\frac{second\;order\;adjusted\;ToM\;response\;time\;score-{RT}_{min}}{{RT}_{i}}}{2}$$

with RT_i_ referring to the total seconds available (RTi = 60) and RT^min^ referring to the minimum seconds available (for first-order items, RT_min_ = 1.50; for second-order items, RT_min_ = 2.54). In addition, two scores are computed to assess the balance between cognitive and affective ToM performance, the Cognitive/Affective Accuracy Index (CA_A_) and the Cognitive/Affective Response Time Index (CA_RT_), through the following formula:

$${CA}_{A}=\frac{affective\;adjusted\;ToM\;accuracy-cognitive\;adjusted\;ToM\;accuracy}{affective\;adjusted\;ToM\;accuracy+cognitive\;adjusted\;ToM\;accuracy}$$

$${CA}_{RT}=\left(affective\;adjusted\;ToM\;response\;time-cognitive\;adjusted\;ToM\;response\;time\right)\times (-1)$$

A CA_A_/CA_RT_ score near 0 indicates a balance between affective and cognitive ToM performance. A score far from 0 suggests a higher (positive score, e.g., 1) or a lower (negative score, e.g., −1) performance of affective than cognitive ToM performance.

Supplementary Materials report the scoring calculator (Data Sheet) and the Yoni-36 task scoring sheet (Table A2).

Adjustment values to compute the ACC and RT score are reported in Table 4 and Table 5.

### 3.6. Normative Data Extraction

Table 6 reports normative data and the equivalence scores extracted, respectively.

## 4. Discussion

The goal of the present study was to investigate the psychometric properties of the Italian 36-item version of the Yoni task [48] to propose a quick and reliable tool for assessing ToM within routine clinical practice. Following earlier studies [48,49], the validity and reliability of the Yoni-36 item were investigated in terms of discrimination ability, internal consistency, and split-half reliability. In addition, correction grids, normative data, and equivalent scores have been provided for the Italian population, contributing to the field of neurocognitive assessment by introducing a ToM screening measure for the diagnostic process in routine clinical practice.

Overall, the Yoni-36 showed good psychometric properties. In line with our previous study [49], the second-order ToM items could distinguish between high and low performance in healthy adults. As expected, low discrimination ability was reported for the first-order ToM items. In fact, while the first-order level of ToM is already consolidated in early childhood [50], mature ToM skills, mainly involving the second-order ToM abilities, grow over the early adulthood period [18]. Nevertheless, neurological conditions, such as dementia, are usually characterized by a neurocognitive deficit extending also to the first-order ToM level [51,52], and the assessment of this ToM domain in the clinical setting might be useful. Concerning the validity of the Yoni-36 task, the internal consistency was high, supporting its adoption as a neurocognitive ToM measure for neuropsychological assessment. Moreover, the test demonstrated a good split-half reliability, in line with previous results [48,49]. Although future studies need to test additional psychometric evidence, such as the test-retest reliability and the diagnostic validity, these findings suggest satisfactory properties of the Yoni-36 task, differently from other common measures. It is worth noting that most ToM measures showed low or sub-optimal psychometric standards, and the validation of further tools or test versions is encouraged [53,54]. In fact, unlike the five traditional neurocognitive domains (learning and memory, executive function, complex attention, perceptual-motor function, and language), whose assessment tools have been subjected to a long history of validation, studies on the psychometric properties and normative data for ToM tests are limited. To our knowledge, correction grids and normative data are available for a limited number of tests, such as the Faux Pas test and Story-based Empathy task [38,45], and the Eyes Test [39] remains the ToM test validated and adapted for different languages and age groups [55,56].

Concerning the influence of demographic variables on the Yoni-36 task scores, we observed that age and education mainly affected both performance and response time, as highlighted in previous studies on the Yoni task [48,49]. Greater performance appeared to be associated with younger age and a greater education level. Altogether, our findings confirm previous evidence on age-related changes across adulthood [57,58] and the role of education as a mediator on the link between age and ToM performance [57]. Considering age, a meta-analysis [59] including data from six types of ToM tasks, different ToM components, and administration modalities (verbal, visuo-spatial, and dynamic) confirmed a decline in ToM in old age, regardless of type of ToM task, component, and stimuli modality. Moving to education, a previous study [60] highlighted the role of education on ToM ability, showing that people with greater educational attainment were more proficient in mentalizing tasks. Age and years of education have been demonstrated to influence the performance of other ToM tools, such as the Edinburgh Social Cognition test [42], the Story-based Empathy Task [38], and the Reading the Mind in the Eyes test [46], commonly used ToM tests in the clinical context. Considering the Yoni-36 task sub-scores, the role of education on all the scores, including the ToM first-order score, was not unexpected. In fact, education attainment as well as age influence brain reserve and resilience and act as a cognitive reserve proxy by modulating the effect of disease-related brain changes on cognitive performance [61,62]. Namely, a high educational level may protect against the age-related ToM decline.

Finally, normative data and equivalent scores were provided for the Yoni-36 task. High accuracy levels, response time, and affective-cognitive ToM balance were observed in healthy Italian subjects. Our computed equivalent scores will allow the comparison between the Yoni-36 task performance and other neuropsychological measures with different natures and metrics [63,64]. Norming ToM measures is essential to make the task suitable for clinical purposes, allowing for an objective judgment regarding a patient’s neurocognitive profile [65]. Additional studies are needed to confirm the diagnostic validity of the Yoni-36, investigating the sensitivity and specificity in discriminating clinical populations with ToM deficits from healthy conditions. Such work will allow for testing the suitability of the Yoni task in specific clinical settings. In fact, although the present study provides a cut-off to identify people with a ToM performance far from the norm, additional contributions on specific clinical populations will allow for a deeper understanding of social cognition phenotypes in clinical profiles.

This study is not without limitations. Further analyses on the validity and reliability of the Yoni task would have strengthened our conclusions. In more detail, testing the divergent validity on spatial perception, reasoning, or face recognition may add information on the relationship between the Yoni task and other cognitive functions. In fact, convergent and divergent validity evidence of the Yoni task has already been presented in previous studies [48,49] only on gender recognition tasks. Moreover, a future inter-time reliability study is needed to support the test adoption for patients undergoing treatment. Finally, it is worth mentioning that the Yoni task presents simple cartoon-like stimuli that do not consist of complex real-life scenarios such as other ToM tests (e.g., the Edinburgh Social Cognition test and the Moving for the Assessment of Social Cognition). Therefore, its application should be targeted towards certain clinical populations in which reduced cognitive load may facilitate a better characterization of specific ToM deficits (e.g., individuals with Alzheimer’s disease) [8].

## 5. Conclusions

In conclusion, we collected a set of norms for the short version of the Yoni task (36 items) in healthy Italian subjects. The Yoni-36 task proved to be a valid instrument for a quick and reliable ToM assessment, suitable to be included in neuropsychological batteries as recommended by the DSM-5-TR [5]. Its good psychometric properties support the appropriateness of the tool in capturing ToM accuracy and response time considering both affective and cognitive aspects, paving the way for further application of the Yoni task in Italian research and clinical contexts. Especially, it constitutes a shorter, valid alternative to the Yoni-48 task, with the advantage of decreasing time required for the administration, which is attractive in the face of time constraints in the clinic. Finally, it may be used as a ToM screening tool, capable of broadly capturing ToM levels and components that can be further investigated as needed with specific tasks.

## Figures and Tables

**Table 1:** Number of subjects for sex, age, and education groups.

Socio-Demographic Variables	Participants		
	Males	Females	Total
*Age group*			
*18–29*	53	54	107
*30–39*	27	30	57
*40–49*	18	22	40
*50–59*	22	26	48
*60–60*	17	20	37
*>70*	15	14	29
*Age years (M, sd)*	42.11 ± 18.25	42.31 ± 18.04	42.22 ± 18.11
*Years of education*			
*<8 years*	18	23	41
*13 years*	60	59	119
*≥16 years*	74	84	158
*Education years (M, sd)*	15.03 ± 3.05	14.99 ± 3.75	15.01 ± 3.43

**Table 2:** Inter-items and split-half reliability of the Yoni-36 task.

	Cronbach’s α	McDonald’s ω	Guttman’s λ^2^	Guttman’s λ^6^	Split-Half Spearman-Brown ϱ_SP_	
					Median	95% HDI
total scale	0.84	0.85	0.86	0.90	0.85	0.77–0.90
second-order items	0.84	0.84	0.85	0.87	0.85	0.75–0.89
first-order items	0.75	0.73	0.77	0.84	0.76	0.63–0.86
affective items	0.71	0.70	0.73	0.76	0.73	0.58–0.80
cognitive items	0.78	0.82	0.82	0.80	0.82	0.70–0.86

**Table 3:** Predictors of the Yoni-36 scores.

	Predictors	*β*	SE	t	Targeted *p*	R^2^	Omnibus *p*
Accuracy							
First–order	Education	0.02	0.03	0.90	0.364	0.01	0.482
	Age	−0.01	0.00	−1.06	0.291		
	Sex	−0.04	0.14	−0.30	0.764		
	Intercept	15.33	0.46	33.29	<0.001		
Second–order	Education	0.20	0.07	3.01	0.003	0.07	<0.001
	Age	−0.03	0.01	−3.45	<0.001		
	Sex	−0.01	0.35	−0.04	0.968		
	Intercept	11.87	1.12	10.60	<0.001		
Affective	Education	0.07	0.04	1.72	0.087	0.04	0.007
	Age	−0.01	0.01	−2.41	0.017		
	Sex	−0.02	0.22	−0.09	0.929		
	Intercept	14.09	0.70	20.22	<0.001		
Cognitive	Education	0.16	0.05	3.31	0.001	0.08	<0.001
	Age	−0.02	0.01	−3.41	<0.001		
	Sex	−0.04	0.25	−0.15	0.878		
	Intercept	13.11	0.78	16.74	<0.001		
RT							
First–order	Education	−0.19	0.06	−3.20	0.002	0.05	0.001
	Age	0.01	0.01	1.63	0.105		
	Sex	0.25	0.32	0.80	0.422		
	Intercept	7.19	0.99	7.19	<0.001		
Second–order	Education	−0.28	0.07	−3.93	<0.001	0.22	<0.001
	Age	0.08	0.01	7.80	<0.001		
	Sex	−0.02	0.38	−0.06	0.949		
	Intercept	9.61	1.20	8.00	<0.001		
Affective	Education	−0.29	0.07	−4.27	<0.001	0.12	<0.001
	Age	0.04	0.01	3.90	<0.001		
	Sex	0.21	0.35	0.58	0.560		
	Intercept	9.72	1.12	8.64	<0.001		
Cognitive	Education	−0.19	0.06	−3.13	0.002	0.17	<0.001
	Age	0.06	0.01	6.64	<0.001		
	Sex	0.02	0.31	0.07	0.943		
	Intercept	7.08	0.99	7.12	<0.001		

**Table 4:** Adjustment values to be subtracted/summed to the accuracy raw scores of the Yoni-36 task.

		*≤8 Years of Education*		*13 Years of Education*		*≥16 Years of Education*	
		*Males*	*Females*	*Males*	*Females*	*Males*	*Females*
	Age groups						
First–Order	18–29	−0.03	−0.07	−0.12	−0.17	−0.19	−0.23
	30–39	0.08	0.04	−0.02	−0.06	−0.08	−0.12
	40–49	0.18	0.14	0.08	0.04	0.02	−0.02
	50–59	0.28	0.24	0.18	0.14	0.12	0.08
	60–69	0.38	0.34	0.28	0.24	0.22	0.18
	≥70	0.48	0.44	0.38	0.34	0.32	0.28
	18–29	0.84	0.83	−0.15	−0.16	−0.75	−0.76
Second–Order	30–39	1.17	1.16	0.17	0.16	−0.42	−0.43
	40–49	1.47	1.46	0.47	0.46	−0.12	−0.13
	50–59	1.77	1.76	0.77	0.76	0.17	0.16
	60–69	2.07	2.06	1.07	1.06	0.47	0.46
	≥70	2.37	2.36	1.37	1.36	0.77	0.76
Affective	18–29	0.31	0.29	−0.04	−0.06	−0.25	−0.27
	30–39	0.42	0.40	0.07	0.05	−0.14	−0.16
	40–49	0.52	0.50	0.17	0.15	−0.04	−0.06
	50–59	0.62	0.60	0.27	0.25	0.06	0.04
	60–69	0.72	0.70	0.37	0.35	0.16	0.14
	≥70	0.82	0.80	0.47	0.45	0.26	0.24
Cognitive	18–29	0.77	0.73	−0.03	−0.07	−0.51	−0.55
	30–39	0.99	0.95	0.19	0.15	−0.30	−0.33
	40–49	1.19	1.15	0.39	0.35	−0.09	−0.13
	50–59	1.39	1.35	0.59	0.55	0.11	0.07
	60–69	1.59	1.55	0.79	0.75	0.31	0.27
	≥70	1.79	1.75	0.99	0.95	0.51	0.47

*Adjustment values for sex, age, and education of the Yoni-36 accuracy raw scores. The upper and lower limit accuracy scores have not to be adjusted.*

**Table 5:** Adjustment values to be subtracted/summed to the response time raw scores of the Yoni-36 task.

		≤8 Years of Education		13 Years of Education		≥16 Years of Education	
		Males	Females	Males	Females	Males	Females
Age groups							
First–Order	18–29	−1.26	−1.01	−0.31	−0.06	0.26	0.51
	30–39	−1.37	−1.12	−0.42	−0.17	0.15	0.40
	40–49	−1.47	−1.22	−0.52	−0.27	0.05	0.30
	50–59	−1.57	−1.32	−0.62	−0.37	−0.05	0.20
	60–69	−1.67	−1.42	−0.72	−0.47	−0.15	0.10
	≥70	−1.77	−1.52	−0.82	−0.57	−0.25	0.00
Second–Order	18–29	−0.53	−0.48	0.76	0.92	1.53	1.76
	30–39	−1.34	−1.36	0.06	0.04	0.90	0.88
	40–49	−2.14	−2.16	−0.74	−0.76	0.10	0.08
	50–59	−2.94	−2.96	−1.54	−1.56	−0.70	−0.72
	60–69	−3.74	−3.76	−2.34	−2.36	−1.50	−1.52
	≥70	−4.54	−4.56	−3.14	−3.16	−2.30	−2.32
Affective	18–29	−1.38	−1.17	0.07	0.28	0.94	1.15
	30–39	−1.82	−1.61	−0.37	−0.16	0.50	0.71
	40–49	−2.22	−2.01	−0.77	−0.56	0.10	0.31
	50–59	−2.62	−2.41	−1.17	−0.96	−0.30	−0.09
	60–69	−3.02	−2.81	−1.57	−1.36	−0.70	−0.49
	≥70	−3.42	−3.21	−1.97	−1.76	−1.10	−0.89
Cognitive	18–29	−0.22	−0.20	0.73	0.75	1.30	1.32
	30–39	−0.88	−0.86	0.07	0.09	0.64	0.66
	40–49	−1.48	−1.46	−0.53	−0.51	0.04	0.06
	50–59	−2.08	−2.06	−1.13	−1.11	−0.56	−0.54
	60–69	−2.68	−2.66	−1.73	−1.71	−1.16	−1.14
	≥70	−3.28	−3.26	−2.33	−2.31	−1.76	−1.74

Adjustment values for sex, age, and education of the Yoni-36 RT raw scores. The upper and lower limit RT scores have not to be adjusted.

**Table 6:** Normative data and equivalent scores of the Yoni-36 task in the Italian population.

	Accuracy	Response Time (s)
*First-order score*		
range score (min, max)	0.00–16.00	0.00–60.00
mean, SD	15.49, 1.28	5.07, 2.81
median	16.00	4.40
25th–75th percentile	16.00–16.00	3.10–6.30
95% CI (lower, upper)	15.49, 15.63	4.76, 5.37
*Second-order score*		
range score (min, max)	0.00–16.00	0.00–60.00
mean, SD	13.29, 3.11	8.84, 3.36
median	14.19	8.33
25th–75th percentile	11.95–16.00	6.43–10.57
95% CI (lower, upper)	13.28, 13.63	8.47, 9.21
*Affective score*		
range score (min, max)	0.00–16.00	0.00–60.00
mean, SD	14.48, 1.96	7.15, 3.15
median	15.02	6.36
25th–75th percentile	13.69–16.00	4.93–8.67
95% CI (lower, upper)	14.27, 14.70	6.80, 7.50
*Cognitive score*		
range score (min, max)	0.00–16.00	0.00–60.00
mean, SD	14.31, 2.18	6.74, 2.78
median	15.23	6.18
25th–75th percentile	13.15–16.00	4.86–8.18
95% CI (lower, upper)	14.07, 14.55	6.44, 7.05
*total score (ACC/RT)*		
range score (min, max)	0.00–1.00	0.00–1.00
mean, SD	0.90, 0.11	0.92, 0.05
median	0.93	0.92
25th–75th percentile	0.84–1.00	0.89–0.95
95% CI (lower, upper)	0.89, 0.91	0.91, 0.92
*CA score*		
range score (min, max)	-	-
mean, SD	0.01, 0.08	−0.40, 2.34
median	0.00	−0.19
25th–75th percentile	−0.03–0.04	−1.57–0.83
95% CI (lower, upper)	−0.01, 0.02	−0.66, −0.14
*Equivalent score*		
4	>0.93	-
3	0.92–0.90	-
2	0.89–0.79	-
1	0.78–0.69	-
0	<0.69	<0.83

Legend: CI, confidence interval; SD, standard deviation; ACC, accuracy ToM score; RT, response time ToM score.

## Data Availability

The raw data supporting the conclusions of this article will be made available upon reasonable request by the corresponding author.

## Supplementary Materials

The following supporting information can be downloaded at https://www.mdpi.com/article/10.3390/jcm13216334/s1.

## Appendix A

**Table A1:** The Yoni-36 task items discrimination ability.

Item n.	ToM Component	ToM Level	RT Raw Score	ACC Raw Score	h
1	Affective	First	5.30	0.93	0.35
2	Cognitive	First	5.38	0.94	0.45
5	Cognitive	First	3.69	0.98	0.40
6	Affective	First	4.19	0.98	0.16
7	Cognitive	First	6.15	0.98	0.44
8	Affective	First	6.46	0.97	0.19
9	Affective	First	4.67	0.96	0.45
10	Affective	First	5.28	0.98	0.40
11	Cognitive	First	4.31	0.99	0.18
12	Cognitive	First	3.53	0.99	0.25
13	Affective	First	8.19	0.93	0.47
14	Affective	Second	11.40	0.76	0.87
15	Affective	First	6.02	0.96	0.36
16	Affective	First	5.67	0.89	0.63
17	Affective	Second	10.50	0.81	0.76
18	Affective	Second	6.61	0.88	0.76
19	Cognitive	First	4.40	0.98	0.44
20	Affective	Second	8.87	0.92	0.74
21	Affective	Second	10.70	0.90	0.77
22	Affective	Second	9.27	0.91	0.76
23	Cognitive	First	5.81	0.98	0.44
24	Cognitive	First	5.43	0.96	0.42
26	Cognitive	Second	10.20	0.78	1.19
27	Cognitive	Second	9.14	0.76	1.07
28	Affective	Second	8.69	0.76	0.99
29	Cognitive	Second	8.47	0.86	1.04
30	Cognitive	Second	7.71	0.88	1.13
31	Cognitive	Second	8.83	0.85	0.96
32	Affective	Second	7.30	0.86	0.96
34	Cognitive	Second	11.20	0.71	1.01
35	Cognitive	Second	9.57	0.66	1.21
36	Cognitive	Second	7.34	0.87	0.90

Items 3, 4, 25, and 33 are not reported in the table as they are control items (physical content).

**Table A2:** Scoring sheet example.

	Accuracy			Response Time	
	Raw Score	Adjusted Score		Raw Score	Adjusted Score
first order ToM (0–16)			first order ToM (0–60)		
second order ToM (0–16)			second order ToM (0–60)		
affective ToM (0–16)			affective ToM (0–60)		
cognitive ToM (0–16)			cognitive ToM (0–60)		
physical (0–4)			physical (0–60)		
ACC (0–1)			RT (0–1)		
CA_A_			CA_RT_		

Legend: ACC, accuracy ToM score; RT, response time ToM score; ToM, Theory of Mind; CA_A_, Cognitive/Affective accuracy index; CA_RT_, Cognitive/Affective response time index.

## References

[1] Frith C.D., Frith U. (2007). Social cognition in humans. Curr. Biol..

[2] Baglio F., Marchetti A. (2016). Editorial: When (and How) Is Theory of Mind Useful? Evidence from Life-Span Research. Front. Psychol..

[3] Beauchamp M.H. (2017). Neuropsychology’s social landscape: Common ground with social neuroscience. Neuropsychology.

[4] Dodich A., Crespi C., Santi G.C., Cappa S.F., Cerami C. (2021). Evaluation of Discriminative Detection Abilities of Social Cognition Measures for the Diagnosis of the Behavioral Variant of Frontotemporal Dementia: A Systematic Review. Neuropsychol. Rev..

[5] Henry J.D., von Hippel W., Molenberghs P., Lee T., Sachdev P.S. (2016). Clinical assessment of social cognitive function in neurological disorders. Nat. Rev. Neurol..

[6] McDonald S., Genova H. (2021). The effect of severe traumatic brain injury on social cognition, emotion regulation, and mood. Handb. Clin. Neurol..

[7] Van den Stock J., Bertoux M., Diehl-Schmid J., Piguet O., Rankin K.P., Pasquier F., Ducharme S., Pijnenburg Y., Kumfor F. (2023). Current Potential for Clinical Optimization of Social Cognition Assessment for Frontotemporal Dementia and Primary Psychiatric Disorders. Neuropsychol. Rev..

[8] Shamay-Tsoory S.G., Aharon-Peretz J. (2007). Dissociable prefrontal networks for cognitive and affective theory of mind: A lesion study. Neuropsychologia.

[9] Kalbe E., Schlegel M., Sack A.T., Nowak D.A., Dafotakis M., Bangard C., Brand M., Shamay-Tsoory S., Onur O.A., Kessler J. (2010). Dissociating cognitive from affective theory of mind: A TMS study. Cortex.

[10] Sebastian C.L., Fontaine N.M., Bird G., Blakemore S.J., Brito S.A., McCrory E.J., Viding E. (2012). Neural processing associated with cognitive and affective Theory of Mind in adolescents and adults. Soc. Cogn. Affect. Neurosci..

[11] Eslinger P.J. (1998). Neurological and neuropsychological bases of empathy. Eur. Neurol..

[12] Baron-Cohen S., Campbell R., Karmiloff-Smith A., Grant J., Walker J. (1995). Are children with autism blind to the mentalistic significance of the eyes?. Br. J. Dev. Psychol..

[13] Wellman H.M., Woolley J.D. (1990). From simple desires to ordinary beliefs: The early development of everyday psychology. Cognition.

[14] Wimmer H., Perner J. (1983). Beliefs about beliefs: Representation and constraining function of wrong beliefs in young children’s understanding of deception. Cognition.

[15] Perner J., Wimmer H. (1985). “John thinks that Mary thinks that…” attribution of second-order beliefs by 5-to 10-year-old children. J. Exp. Child Psychol..

[16] Shamay-Tsoory S.G., Tomer R., Berger B.D., Goldsher D., Aharon-Peretz J. (2005). Impaired “affective theory of mind” is associated with right ventromedial prefrontal damage. Cogn. Behav. Neurol..

[17] Ruitenberg M.F.L., Santens P., Notebaert W. (2020). Cognitive and Affective Theory of Mind in Healthy Aging. Exp. Aging Res..

[18] Bianco F., Castelli I. (2023). The promotion of mature theory of mind skills in educational settings: A mini-review. Front. Psychol..

[19] Bottiroli S., Cavallini E., Ceccato I., Vecchi T., Lecce S. (2016). Theory of Mind in aging: Comparing cognitive and affective components in the faux pas test. Arch. Gerontol. Geriatr..

[20] Abu-Akel A., Shamay-Tsoory S. (2011). Neuroanatomical and neurochemical bases of theory of mind. Neuropsychologia.

[21] d‘Arma A., Isernia S., Di Tella S., Rovaris M., Valle A., Baglio F., Marchetti A. (2021). Social Cognition Training for Enhancing Affective and Cognitive Theory of Mind in Schizophrenia: A Systematic Review and a Meta-Analysis. J. Psychol..

[22] Isernia S., Baglio F., d‘Arma A., Groppo E., Marchetti A., Massaro D. (2019). Social Mind and Long-Lasting Disease: Focus on Affective and Cognitive Theory of Mind in Multiple Sclerosis. Front. Psychol..

[23] Isernia S., Cabinio M., Pirastru A., Mendozzi L., Di Dio C., Marchetti A., Massaro D., Baglio F. (2020). Theory of mind network in multiple Sclerosis: A double disconnection mechanism. Soc. Neurosci..

[24] Roca M., Manes F., Gleichgerrcht E., Ibáñez A., González de Toledo M.E., Marenco V., Bruno D., Torralva T., Sinay V. (2014). Cognitive but not affective theory of mind deficits in mild relapsing-remitting multiple sclerosis. Cogn. Behav. Neurol..

[25] Coundouris S.P., Adams A.G., Henry J.D. (2020). Empathy and theory of mind in Parkinson’s disease: A meta-analysis. Neurosci. Biobehav. Rev..

[26] Maggi G., Cima Muñoz A.M., Obeso I., Santangelo G. (2022). Neuropsychological, neuropsychiatric, and clinical correlates of affective and cognitive theory of mind in Parkinson’s disease: A meta-analysis. Neuropsychology.

[27] Rossetto F., Castelli I., Baglio F., Massaro D., Alberoni M., Nemni R., Shamay-Tsoory S., Marchetti A. (2018). Cognitive and Affective Theory of Mind in Mild Cognitive Impairment and Parkinson’s Disease: Preliminary Evidence from the Italian Version of the Yoni Task. Dev. Neuropsychol..

[28] Laisney M., Bon L., Guiziou C., Daluzeau N., Eustache F., Desgranges B. (2013). Cognitive and affective Theory of Mind in mild to moderate Alzheimer’s disease. J. Neuropsychol..

[29] Rossetto F., Isernia S., Cabinio M., Pirastru A., Blasi V., Baglio F. (2022). Affective Theory of Mind as a residual ability to preserve mentalizing in amnestic Mild Cognitive Impairment: A 12-months longitudinal study. Front. Neurol..

[30] Roheger M., Grothe L., Hasselberg L., Grothe M., Meinzer M. (2024). A systematic review and meta-analysis of socio-cognitive impairments in multiple sclerosis. Sci. Rep..

[31] Cavallini E., Lecce S., Bottiroli S., Palladino P., Pagnin A. (2013). Beyond false belief: Theory of mind in young, young-old, and old-old adults. Int. J. Aging Hum. Dev..

[32] Kemp J., Després O., Sellal F., Dufour A. (2012). Theory of Mind in normal ageing and neurodegenerative pathologies. Ageing Res. Rev..

[33] Poletti M., Bonuccelli U. (2013). Alteration of affective Theory of Mind in amnestic mild cognitive impairment. J. Neuropsychol..

[34] Adenzato M., Poletti M. (2013). Theory of Mind abilities in neurodegenerative diseases: An update and a call to introduce mentalizing tasks in standard neuropsychological assessments. Clin. Neuropsychiaty.

[35] Baron-Cohen S., Leslie A.M., Frith U. (1985). Does the autistic child have a “theory of mind”?. Cognition.

[36] Stone V.E., Baron-Cohen S., Knight R.T. (1998). Frontal lobe contributions to theory of mind. J. Cogn. Neurosci..

[37] Happé F.G., Winner E., Brownell H. (1998). The getting of wisdom: Theory of mind in old age. Dev. Psychol..

[38] Dodich A., Cerami C., Canessa N., Crespi C., Iannaccone S., Marcone A., Realmuto S., Lettieri G., Perani D., Cappa S.F. (2015). A novel task assessing intention and emotion attribution: Italian standardization and normative data of the Story-based Empathy Task. Neurol. Sci..

[39] Baron-Cohen S., Wheelwright S., Hill J., Raste Y., Plumb I. (2001). The “Reading the Mind in the Eyes” Test revised version: A study with normal adults, and adults with Asperger syndrome or high-functioning autism. J. Child. Psychol. Psychiatry.

[40] McDonald S., Flanagan S., Martin I., Saunders C. (2004). The ecological validity of TASIT: A test of social perception. Neuropsychol. Rehabil..

[41] Baksh R.A., Abrahams S., Auyeung B., MacPherson S.E. (2018). The Edinburgh Social Cognition Test (ESCoT): Examining the effects of age on a new measure of theory of mind and social norm understanding. PLoS ONE.

[42] Isernia S., MacPherson S.E., Baksh R.A., Bergsland N., Marchetti A., Baglio F., Massaro D. (2022). Italian adaptation of the Edinburgh Social Cognition Test (ESCoT): A new tool for the assessment of theory of mind and social norm understanding. Front. Psychol..

[43] Isernia S., Pirastru A., Rossetto F., Cacciatore D.M., Cazzoli M., Blasi V., Baksh R.A., MacPherson S.E., Baglio F. (2024). Human reasoning on social interactions in ecological contexts: Insights from the theory of mind brain circuits. Front. Neurosci..

[44] Dziobek I., Fleck S., Kalbe E., Rogers K., Hassenstab J., Brand M., Kessler J., Woike J.K., Wolf O.T., Convit A. (2006). Introducing MASC: A movie for the assessment of social cognition. J. Autism Dev. Disord..

[45] Delgado-Álvarez A., Pytel V., Delgado-Alonso C., Olbrich-Guzmán C.M., Cortés-Martínez A., Moreno-Ramos T., Montero-Escribano P., Matías-Guiu J., Matias-Guiu J.A. (2021). Development, Spanish Normative Data, and Validation of a Social Cognition Battery in Prodromal Alzheimer’s Disease and Multiple Sclerosis. Arch. Clin. Neuropsychol..

[46] Maddaluno O., Aiello E.N., Roncoroni C., Prunas A., Bolognini N. (2022). The Reading the Mind in the Eyes Test, Iowa Gambling Task and Interpersonal Reactivity Index: Normative Data in an Italian Population Sample. Arch. Clin. Neuropsychol..

[47] Gourlay C., Collin P., Caron P.O., D’Auteuil C., Scherzer P.B. (2022). Psychometric assessment of social cognitive tasks. Appl. Neuropsychol. Adult.

[48] Isernia S., Rossetto F., Blasi V., Massaro D., Castelli I., Ricci C., Shamay-Tsoory S., Marchetti A., Baglio F. (2022). Measuring cognitive and affective Theory of Mind with the Italian Yoni task: Normative data and short versions. Curr. Psychol..

[49] Isernia S., Rossetto F., Shamay-Tsoory S., Marchetti A., Baglio F. (2022). Standardization and normative data of the 48-item Yoni short version for the assessment of theory of mind in typical and atypical conditions. Front. Aging Neurosci..

[50] Wellman H.M. (2018). Theory of mind: The state of the art. Eur. J. Dev. Psychol..

[51] Rowse G., McCarthy-Jones S., Knowles R., Corcoran R., Bentall R.P. (2013). Attributional style and theory of mind in people with Alzheimer disease and persecutory delusions. Am. J. Geriatr. Psychiatry.

[52] Sava A.A., Delphin-Combe F., Krolak-Salmon P., Michael G.A., Chainay H. (2019). First-order Affective Theory of Mind in Persons with Alzheimer’s Disease and in Healthy Older Adults. Can. J. Aging.

[53] Chen K.W., Lee S.C., Chiang H.Y., Syu Y.C., Yu X.X., Hsieh C.L. (2017). Psychometric properties of three measures assessing advanced theory of mind: Evidence from people with schizophrenia. Psychiatry Res..

[54] Yeh Y.C., Lin C.Y., Li P.C., Hung C.F., Cheng C.H., Kuo M.H., Chen K.L. (2021). A Systematic Review of the Current Measures of Theory of Mind in Adults with Schizophrenia. Int. J. Environ. Res. Public Health.

[55] Vellante M., Baron-Cohen S., Melis M., Marrone M., Petretto D.R., Masala C., Preti A. (2013). The “Reading the Mind in the Eyes” test: Systematic review of psychometric properties and a validation study in Italy. Cogn. Neuropsychiatry.

[56] Fernández-Abascal E.G., Cabello R., Fernández-Berrocal P., Baron-Cohen S. (2013). Test-retest reliability of the Reading the Mind in the Eyes’ test: A one-year follow-up study. Mol. Autism.

[57] Raimo S., Cropano M., Roldán-Tapia M.D., Ammendola L., Malangone D., Santangelo G. (2022). Cognitive and Affective Theory of Mind across Adulthood. Brain Sci..

[58] Rosi A., Cavallini E., Bottiroli S., Bianco F., Lecce S. (2016). Promoting theory of mind in older adults: Does age play a role?. Aging Ment. Health.

[59] Henry J.D., Phillips L.H., Ruffman T., Bailey P.E. (2013). A meta-analytic review of age differences in theory of mind. Psychol. Aging.

[60] Li X., Wang K., Wang F., Tao Q., Xie Y., Cheng Q. (2013). Aging of theory of mind: The influence of educational level and cognitive processing. Int. J. Psychol..

[61] Stern Y., Arenaza-Urquijo E.M., Bartrés-Faz D., Belleville S., Cantilon M., Chetelat G., Ewers M., Franzmeier N., Kempermann G., Kremen W.S. (2020). Whitepaper: Defining and investigating cognitive reserve, brain reserve, and brain maintenance. Alzheimers Dement..

[62] Stern Y., Albert M., Barnes C.A., Cabeza R., Pascual-Leone A., Rapp P.R. (2023). A framework for concepts of reserve and resilience in aging. Neurobiol. Aging.

[63] Capitani E., Laiacona M. (1997). Composite neuropsychological batteries and demographic correction: Standardization based on equivalent scores, with a review of published data. The Italian Group for the Neuropsychological Study of Ageing. J. Clin. Exp. Neuropsychol..

[64] Capitani E., Laiacona M. (2017). Outer and inner tolerance limits: Their usefulness for the construction of norms and the standardization of neuropsychological tests. Clin. Neuropsychol..

[65] Aiello E.N., Depaoli E.G. (2022). Norms and standardizations in neuropsychology via equivalent scores: Software solutions and practical guides. Neurol. Sci..

